# Relationship between oxygen partial pressure and inhibition of cell aggregation of human adipose tissue-derived mesenchymal stem cells stored in cell preservation solutions

**DOI:** 10.1016/j.reth.2023.05.002

**Published:** 2023-06-01

**Authors:** Takeshi Kikuchi, Masuhiro Nishimura, Chikage Shirakawa, Yasutaka Fujita, Takeshige Otoi

**Affiliations:** a Research and Development Center, Otsuka Pharmaceutical Factory, Inc., Naruto, Tokushima, 772-8601, Japan; b Bio-Innovation Research Center, Tokushima University, Myozai-gun, Tokushima, 779-3233, Japan

**Keywords:** Human adipose tissue–derived mesenchymal stem cell, Cell preservation solution, Cell aggregation, Oxygen partial pressure

## Abstract

**Introduction:**

This study investigated the storage conditions under which cell aggregation occurs and the conditions that inhibit cell aggregation when human adipose tissue-derived mesenchymal stem cells (hADSCs) are stored in lactated Ringer’s solution (LR) supplemented with 3% trehalose and 5% dextran 40 (LR-3T-5D).

**Methods:**

We first examined the effects of storage temperature and time on the aggregation and viability of hADSCs stored in LR and LR-3T-5D. The cells were stored at 5 °C or 25 °C for various times up to 24 h. We then evaluated the effects of storage volume (250-2,000 μL), cell density (2.5-20 × 10^5^ cells/mL), and nitrogen gas replacement on aggregation, oxygen partial pressure (pO_2_), and viability of hADSCs stored for 24 h at 25 °C in LR-3T-5D.

**Results:**

When stored in LR-3T-5D, viability did not change under either condition compared with pre-storage, but the cell aggregation rate increased significantly with storage at 25 °C for 24 h (p<0.001). In LR, the aggregation rate did not change under either condition, but cell viability decreased significantly after 24 h at both 5 °C and 25 °C (p < 0.05). The cell aggregation rates and pO_2_ tended to decrease with increasing solution volume and cell density. Nitrogen gas replacement significantly decreased the cell aggregation rate and pO_2_ (p < 0.05). However, there were no differences in viability among cells stored under conditions of different storage volumes, densities, and nitrogen gas replacement.

**Conclusions:**

Aggregation of cells after storage at 25 °C in LR-3T-5D may be suppressed by increasing the storage volume and cell density as well as by incorporating nitrogen replacement, which lowers the pO_2_ in the solution.

## Highlights

• Storing hADSCs in LR-3T-5D at 25 °C for 24 h results in agglomeration.
• Lowering oxygen partial pressure in the solution suppresses cell aggregation.
• Increasing solution volume and cell concentration lowers oxygen partial pressure.
• Nitrogen gas replacement before storage lowers oxygen partial pressure.

## 1 Introduction

Stem cell transplantation is a promising therapy for various diseases, such as Coronavirus Infectious Disease–2019 (COVID-19)–related Acute Respiratory Distress Syndrome [1], cardiovascular disorders [2], autoimmune diseases [3], osteoarthritis [4], liver disorders [5], and graft-versus-host disease [6]. Among stem cells, mesenchymal stem cells (MSCs) are attractive because of their multi-potency in differentiation, immunosuppressive effects, and remodeling effects on extracellular matrices. For intravascular transplantation, cells are often suspended in electrolyte solutions, such as normal saline or saline containing albumin or dextrose [[7], [8], [9], [10]]. However, these solutions are not necessarily ideal for maintaining cell viability and preventing the sedimentation of cells during storage and infusion. Therefore, we developed a better cell preservation solution in which cells can be suspended and stored under refrigeration or at room temperature until use [11].

Severe cell embolization in the lungs can be fatal [12]. It is believed that the risk of vascular embolization increases when a large number of cells are rapidly administered into a blood vessel as part of cell therapy; therefore, preventative measures are generally taken during the procedures, such as administering cells slowly [10]. Indeed, some intravenously administered cell preparations currently on the market prescribe a slow rate of cell administration. In addition to the effect of administration rate, the risk of embolization is elevated when administering populations of large cells, such as pancreatic islets [13]. Because adherent cells naturally exhibit a potential to adhere to each other and form large cell populations, cell aggregation should be considered when administering adherent cells via blood vessels.

Generally, cells or tissues that need to be preserved are kept at a low temperature. However, when cells are administered to patients via infusion bags or syringes, they are maintained at room temperature. As mentioned above, cell administration requires a long time because the cells are administered slowly in order to prevent embolization. Under such conditions, cell storage and administration solutions must maintain the viability of the cells not only at low temperatures but also at room temperature [11].

To date, there have been no reported studies examining the occurrence and inhibition of MSC aggregation under room temperature storage and administration conditions. Therefore, we examined the storage conditions under which aggregation occurs and the conditions that inhibit aggregation when human adipose tissue-derived mesenchymal stem cells (hADSCs) are stored in a solution developed for storage at room temperature.

## 2 Methods

### 2.1 Preservation solutions

Lactated Ringer’s solution (LR; Lactec® Injection) and lactated Ringer’s solution with 3% trehalose and 5% dextran 40 (LR-3T-5D; Cellstor-S) were supplied by Otsuka Pharmaceutical Factory, Inc. (Tokushima, Japan). The LR-3T-5D was developed as a cell-preservation solution in which cells can be suspended and stored under refrigeration or at room temperature [11].

### 2.2 Preparation of adipose tissue-derived mesenchymal stem cells

hADSCs (female, 38Y and 44Y, PT5006, lot nos. 0000421627 and 0000692059, respectively; Lonza Walkersville, Inc., Walkersville, MD, USA) were used in this study; 38Y and 44Y indicate the donors’ age in years. The hADSCs were seeded in a 75 cm^2^ flask with 15 mL of medium prepared from a medium kit (PT-4505 ADSC BulletKit™, Lonza Walkersville, Inc.) and maintained at 37 °C in a humidified 5% CO_2_ atmosphere. The medium was changed every 3 or 4 days; the cells were passaged at approximately 90% confluency, and passages 3, 4, or 5 (4, 5, or 6 after cell preparation) were used for experiments. Cells were washed with phosphate-buffered saline without calcium and magnesium and trypsinized with trypsin/EDTA solution (CC-5012, Lonza Walkersville, Inc.) for 3-5 min at 37 °C. The trypsin reaction was stopped by addition of trypsin-neutralizing solution (CC-5002, Lonza Walkersville, Inc.), and hADSCs were subsequently detached by pipetting. The supernatant containing hADSCs was transferred to a 50 mL conical tube and centrifuged at 210 × *g* for 5 min at room temperature. The supernatant was aspirated, and hADSCs were resuspended in LR for use in storage experiments.

### 2.3 Cell aggregation

**Fig. 1:**
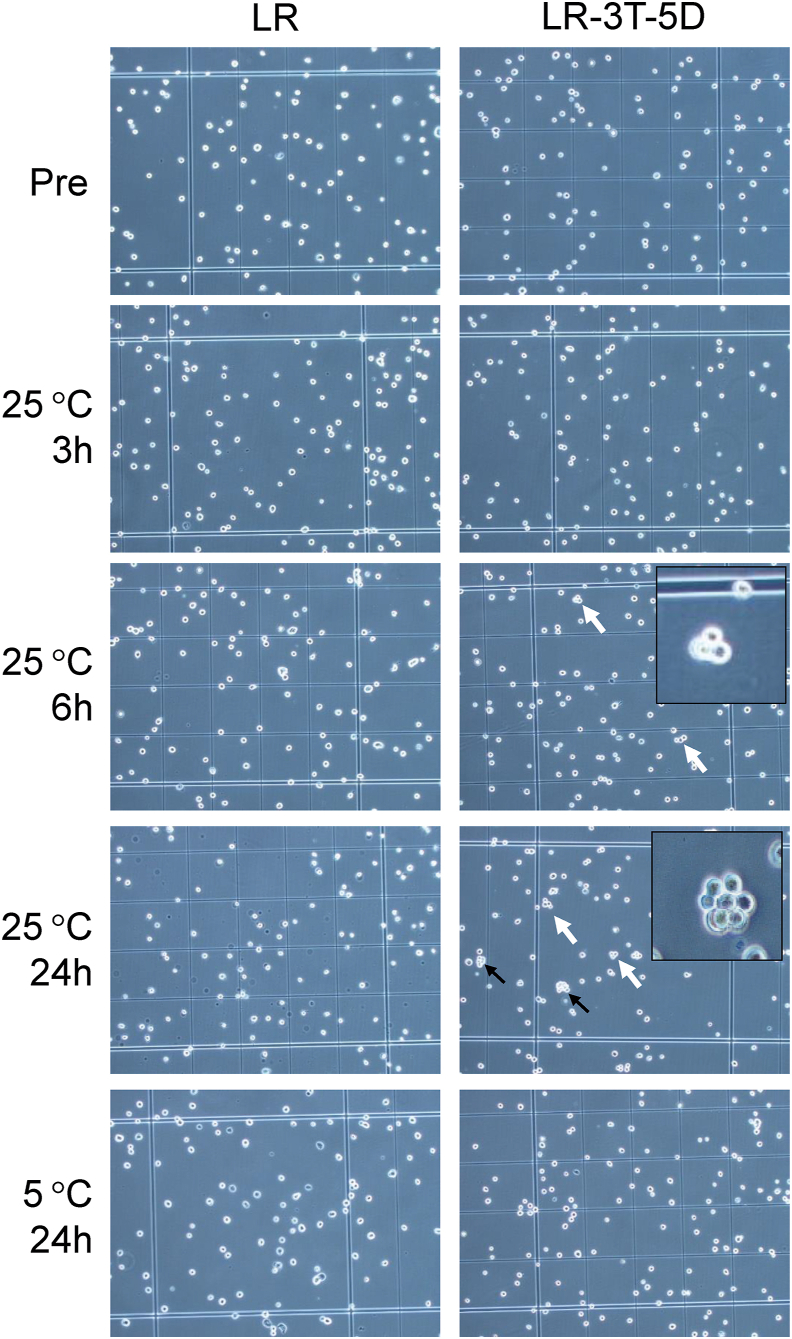
Micrographs of human adipose tissue–derived stem cells (hADSCs) after storage at 5 °C 24 h and 25 °C for each hour in lactated Ringer’s solution (LR) and LR with 3% trehalose and 5% dextran 40 (LR-3T-5D) (magnification 100×). Black arrows indicate cell aggregates (≥5 cells) and white arrows indicate cell clusters of 4 ≤ cells. Inset shows a magnified image of the aggregated cells and cell clusters of 4 ≤ cells.

After storage, samples of cells in the tubes were slowly but thoroughly re-suspended. The total numbers of cells and cell aggregates were determined using a NucleoCounter® NC-200™ (ChemoMetec A/S, Allerød, Denmark). Cell aggregation was defined as an association of ≥5 cells (Fig. 1). This is because it is difficult to measure more than 5 cells in a three-dimensional cell mass. The cell aggregation rate (%) was calculated from the total number of cells counted.

### 2.4 Cell viability

After storage, cells were stained with trypan blue to assess viability. The total numbers of cells and dead cells were determined manually using a plastic cell-counting plate (OneCell Counter, Bio Medical Science, Ltd., Tokyo, Japan). Cell aggregates were excluded from the cell counts. Cell viability was calculated according to the following formula:

Cell viability [%] = (total number of cells − number of dead cells)/(total number of cells) × 100.

### 2.5 Oxygen partial pressure (pO_2_) in storage solution

After storage, the cells in each tube were not re-suspended prior to pO_2_ measurement, which was carried out immediately after sampling according to a standard method using a RAPIDLab348EX Blood Gas System analyzer (Siemens Healthcare K.K., Japan).

### 2.6 Experimental design

The present study was approved by the Ethics Committee of Otsuka Pharmaceutical Factory, Inc. The first experiment examined the effects of storage temperature and time on the aggregation and viability of hADSCs stored in LR and LR-3T-5D. After centrifugation at 210 × *g* for 5 min, the cells were re-suspended at a density of 5 × 10^5^ cells/mL using either LR or LR-3T-5D, and 500 μL of each cell suspension was transferred to low–cell adsorption tubes (STEMFULL™, Sumitomo Bakelite Co., Ltd., Tokyo, Japan). The tubes (15 mL) were tightly capped and stored at 5 °C in a refrigerator or at 25 °C in an incubator for various times up to 24 h.

The second experiment examined the effect of storage volume on aggregation, pO_2_, and viability of hADSCs stored with LR-3T-5D, which was able to maintain viability at 25 °C for 24 h. As described above, the cells were re-suspended in LR-3T-5D at a density of 5 × 10^5^ cells/mL, and 250, 500, 1,000, and 2,000 μL of cell suspension was transferred to separate tubes and stored for 24 h at 25 °C in an incubator.

The third experiment examined the effect of cell density on the aggregation, pO_2_, and viability of hADSCs stored in LR-3T-5D. As described above, the cells were re-suspended in LR-3T-5D at densities of 2 × 10^6^, 1 × 10^6^, 5 × 10^5^, and 2.5 × 10^5^ cells/mL, and 500 μL of cell suspension was transferred to separate tubes and stored for 24 h at 25 °C in an incubator.

The fourth experiment examined the effect of nitrogen gas replacement on the aggregation, pO_2_, and viability of hADSCs. As described above, the cells were re-suspended in LR-3T-5D at a density of 5 × 10^5^ cells/mL, and 250 μL of each cell suspension was transferred to separate tubes. The solution volume was selected as that most likely to cause aggregation based on a prior experiment. A sufficient amount of filter-sterilized nitrogen gas was blown into the sample tubes for 5 s, and then the tubes were tightly capped and stored for 24 h at 25 °C in an incubator. In all experiments, cell samples in tubes were stored under static condition.

### 2.7 Data analysis and statistics

Data are presented as the mean ± standard deviation (SD). Statistical analysis was performed using two-tailed tests, consisting of either a Dunnett’s multiple comparison test or a non-paired Student’s *t*-test. Data were analyzed using SAS 9.4 software (SAS Institute, Inc., Cary, NC, USA).

## 3 Results

### 3.1 Effects of storage temperature and time on the aggregation and viability of hADSCs stored in LR and LR-3T-5D

**Fig. 2:**
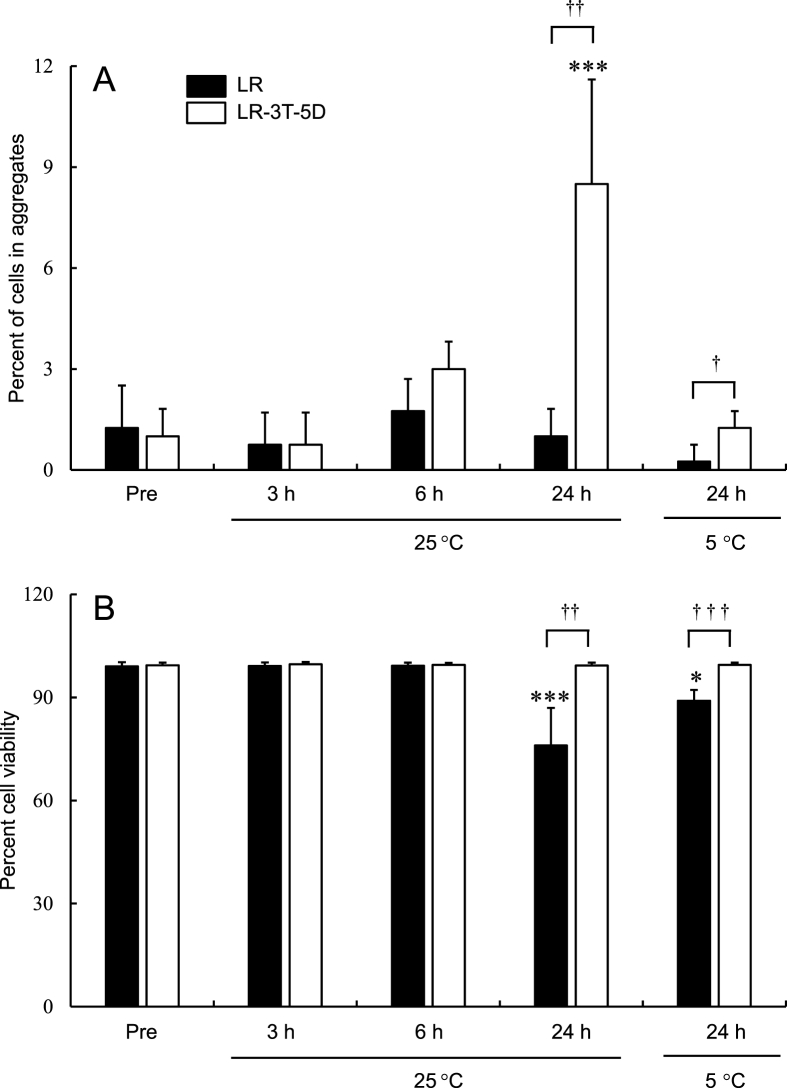
Aggregation rate (A) and viability (B) of hADSCs after storage at 5 °C and 25 °C for various times in lactated Ringer’s solution (LR) and LR-3T-5D. Data are presented as the mean ± SD (n = 4). Statistical analysis was performed using two-tailed Dunnett’s test vs. before storage (Pre): ∗p < 0.05, ∗∗∗p < 0.001, and using two-tailed non-paired *t*-test: †p < 0.05, ††p < 0.01, †††p < 0.001.

As shown in Fig. 2A, there were no significant differences in the aggregation rates of hADSCs stored in LR at 5 °C and 25 °C for up to 24 h compared with pre-storage rates (Pre). However, the aggregation rate of hADSCs stored in LR-3T-5D at 25 °C for 24 h was significantly higher (p < 0.001) than the pre-storage rate. When hADSCs were stored at 25 °C for up to 6 h, the aggregation rate did not differ between cells stored in LR versus LR-3T-5D, but the aggregation rate was significantly higher (p < 0.05) for cells stored in LR-3T-5D than that of cells stored in LR at either 25 °C or 5 °C for 24 h.

The viability of hADSCs stored in LR at 5 °C or 25 °C for 24 h was significantly lower (p < 0.05) than the pre-storage viability, but the viability of cells stored in LR-3T-5D was comparable to the pre-storage viability (Fig. 2B). In addition, the viability of hADSCs stored at 25 °C for up to 6 h did not differ between cells stored in LR versus LR-3T-5D, but the viability of cells stored in LR-3T-5D at either 25 °C or 5 °C for 24 h was significantly higher (p < 0.01) than that of cells stored in LR.

### 3.2 Effect of storage volume on the aggregation, pO_2_, and viability of hADSCs

**Fig. 3:**
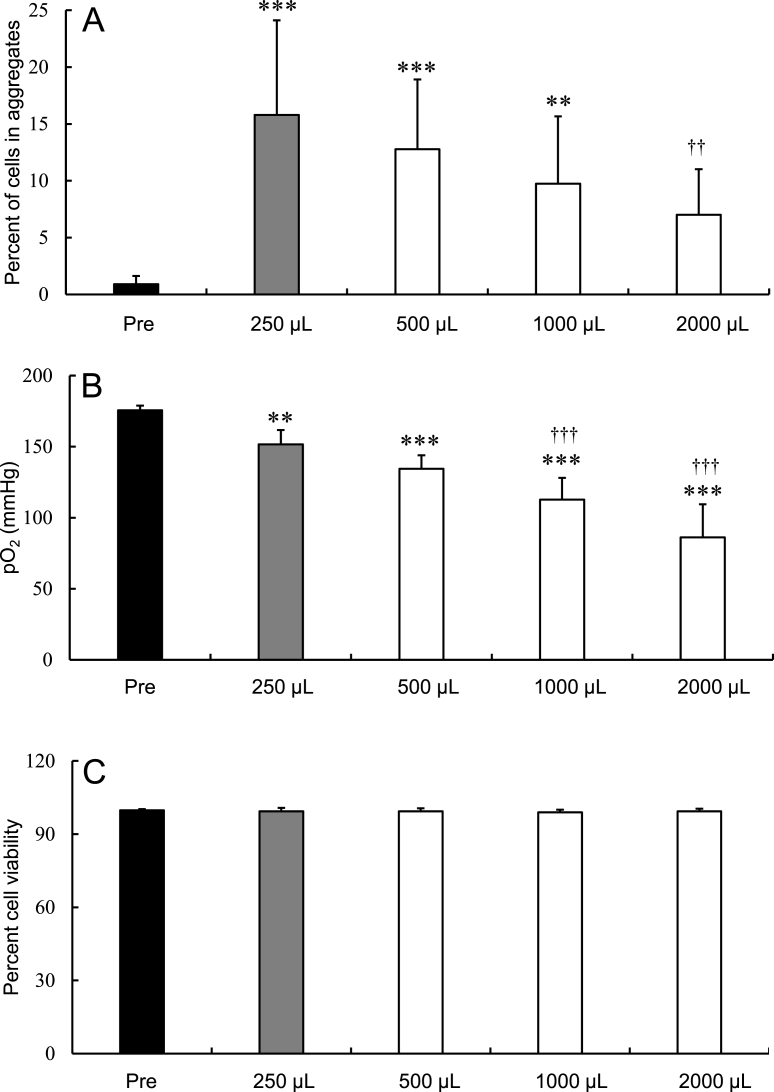
Aggregation rate (A), oxygen partial pressure (pO_2_) in the preservation solution (B), and viability (C) of hADSCs after storage at 25 °C for 24 h in various volumes of LR-3T-5D. Data are presented as the mean ± SD (n = 8–14). Statistical analysis was performed using two-tailed Dunnett’s test vs. before storage (Pre): ∗∗p < 0.01, ∗∗∗p < 0.001, and vs. 250 μL: ††p < 0.01, †††p < 0.001.

When hADSCs (5 × 10^5^ cells/mL) were stored at 25 °C for 24 h in various volumes (250-2000 μL) of LR-3T-5D, the aggregation rate tended to decrease with increasing solution volume (Fig. 3A). The aggregation rates of cells stored in 250, 500, and 1000 μL of LR-3T-5D were significantly higher (p < 0.01) than the pre-storage rates. Moreover, the aggregation rate in 2,000 μL was significantly lower (p < 0.01) than that of cells stored in 250 μL of LR-3T-5D. The post-storage pO_2_ was significantly lower (p < 0.01) than that before storage, irrespective of storage volume (Fig. 3B). Moreover, the pO_2_ tended to decrease with increasing solution volume. The pO_2_ was significantly lower (p < 0.001) in cell samples stored in 1,000 μL and 2,000 μL of LR-3T-5D than in cell samples stored in 250 μL. There were no significant differences in cell viability among the different storage volumes (Fig. 3C).

### 3.3 Effect of cell density on the aggregation, pO_2_, and viability of hADSCs

**Fig. 4:**
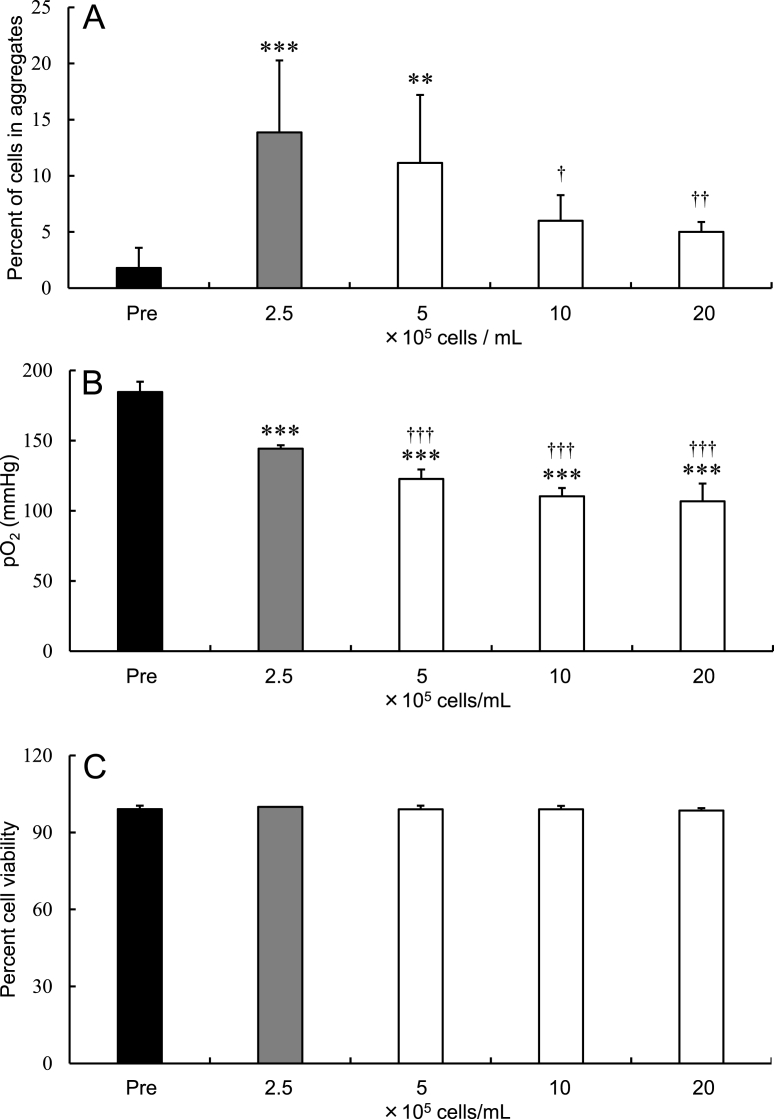
Aggregation rate (A), pO_2_ in the preservation solution (B), and viability (C) of hADSCs after storage at various cell densities at 25 °C for 24 h in LR-3T-5D. Data are presented as the mean ± SD (n = 5–7). Statistical analysis was performed using two-tailed Dunnett’s test before storage (Pre): ∗∗p < 0.01, ∗∗∗p < 0.001, and vs. 2.5 × 10^5^ cells/mL: †p < 0.05, ††p < 0.01, †††p < 0.001.

When hADSCs were stored in LR-3T-5D at 25 °C for 24 h at various densities (2.5-20 × 10^5^ cells/mL), the cell aggregation rate tended to decrease with increasing cell density (Fig. 4A). Compared with the pre-storage rates, the post-storage cell aggregation rates were significantly higher (p < 0.01) for samples stored at densities <5 × 10^5^ cells/mL. Moreover, the cell aggregation rates decreased at densities >10 × 10^5^ cells/mL compared with 2.5 × 10^5^ cells/mL. The post-storage pO_2_ was significantly lower (p < 0.001) than that before storage, irrespective of cell density. Compared with a density of 2.5 × 10^5^ cells/mL, the pO_2_ was significantly lower (p < 0.001) at densities >5 × 10^5^ cells/mL (Fig. 4B). There were no significant differences in viability among cells stored at different densities (Fig. 4C).

### 3.4 Effect of nitrogen gas replacement on the aggregation, pO_2_, and viability of hADSCs

**Fig. 5:**
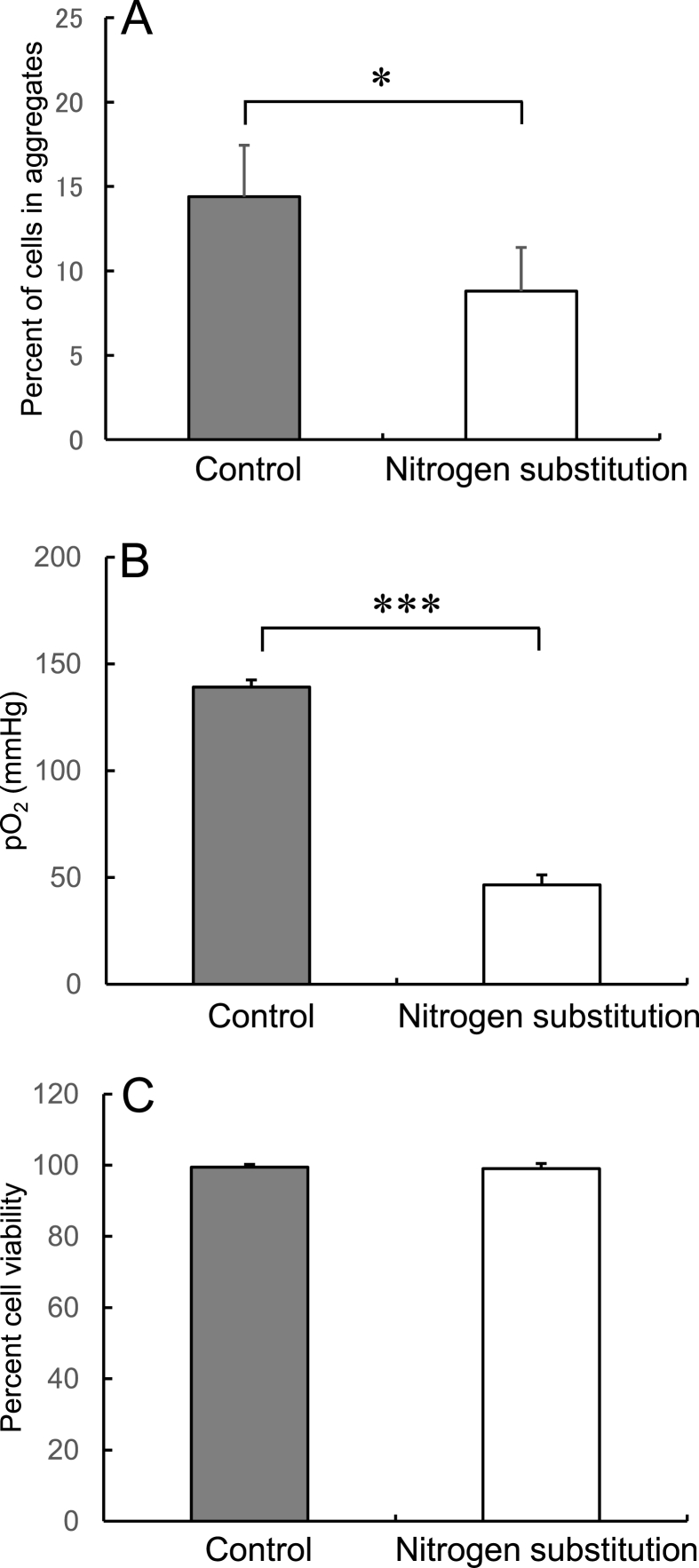
Aggregation rate (A), pO_2_ in the preservation solution (B), and viability (C) of hADSCs after storage at 25 °C for 24 h in LR-3T-5D in tubes with or without (control) nitrogen gas in the head space. Data are presented as the mean ± SD (n = 5). Statistical analysis was performed using two-tailed non-paired *t*-test: ∗p < 0.05, ∗∗∗p < 0.001.

Replacement of the head gas in sample tubes with nitrogen significantly decreased the cell aggregation rate and reduced the pO_2_ from a mean of 139.2 mmHg to 46.6 mmHg (p < 0.05) (Fig. 5A and B). However, there was no difference in viability between hADSCs stored with versus without nitrogen gas replacement (Fig. 5C).

## 4 Discussion

MSCs are often administered intravascularly for cell therapy purposes [[2], [3], [4],^6^,^7^,^9^,^10^], but aggregation can lead to vascular embolization. In the present study, we assessed the effect of various storage conditions on the aggregation of MSCs and provide suggestions regarding how to avoid such aggregation. A high rate of hADSC aggregation was observed when cells were stored in LR-3T-5D at 25 °C for 24 h. Similarly, when human bone marrow-derived mesenchymal stem cells (hBM-MSCs) were stored for 24 h at 25 °C, the aggregation was observed in LR-3T-5D (data not shown). Regardless of origin, MSCs are adhesive cells. Low-adhesive containers were used in storage experiments to avoid adherence to containers and increase the number of cells recovered. On the other hand, MSCs also adhere to each other and form spheroids when cultured in low-adhesion containers [14]. In this experiment, MSCs may have adhered to each other during storage, causing aggregation. In contrast, the aggregation rate of hADSCs stored at 5 °C for 24 h was similar to the pre-storage rate. Typically, cells are stored at low temperatures to inhibit cell metabolism and prevent cell death due to ATP depletion [15]. Moreover, the expression of membrane proteins such as cadherins is reportedly important for MSC adhesion [16]. Although the mechanism of storage-associated cell aggregation remains unclear, aggregation may be inhibited by the suppression of membrane protein function at low temperatures.

In the present study, the aggregation rate and viability of cells stored at 25 °C for up to 6 h were comparable between cells stored in LR versus LR-3T-5D. However, when the cells were stored for 24 h at 25°C, both the aggregation rate and viability of cells stored in LR were lower compared with cells stored in LR-3T-5D. Similar results were observed for cells stored at 5 °C for 24 h. Colony-forming assays have clearly shown that cells with low viability also exhibit low rates of adhesion and proliferation [17]. Our observations also suggest that the decrease in cell viability during storage plays a role in lowering the rate of cell aggregation. In addition, LR-3T-5D contains dextran 40 in order to float the cells in suspension [11]. Dextran coatings reportedly enhance the adhesion of BM-MSCs to sponge scaffolds [18]. Dextran 40 may have been involved in promoting aggregation.

In the present study, we show that the pO_2_ of the preservation solution is related to the rate of cell aggregation after preservation. The following three conditions can be taken to reduce the pO_2_ of the solution and inhibit aggregation without reducing cell viability. First, as shown in Fig. 3A and B, increasing the volume of the preservation solution lowers the pO_2_ in the suspension and the cell aggregation rate. This is a reasonable method that requires no any special manipulation or preparation of a large number of cells. We hypothesize that the pO_2_ in the solution decreases because the volume of the air layer in the tube and the gas exchange efficiency from the solution surface decrease when the volume of solution increases. Second, as shown in Fig. 4A and B, increasing the amount of the preserved cell lowers the pO2 in the suspension and the cell aggregation rate. This is a reasonable method that requires no any special manipulation and without changing the suspension volume. Preserving cells as higher-density suspensions decreases the pO_2_, presumably as a result of increased oxygen consumption due to the increased number of cells in the suspension. Third, as shown in Fig. 5A and B, replacing the head space in the sealed test tube with nitrogen gas was shown to decrease the pO_2_ in the solution and the cell aggregation rate. This method is useful when the number of cells and the volume of the suspension should not be changed. It is assumed that this effect inferred as resulting from the equilibration of oxygen in the solution with the nitrogen in the head space.

In studies using cancer cells, hypoxia has been shown to suppress the expression of cell surface proteins such as cadherins [19,20]. In the present study, nitrogen gas replacement significantly inhibited cell aggregation by reducing the pO_2_ of the preservation solution to 46.6 mmHg after 24 h of storage. The pO_2_ in normal adipose tissue is approximately 50 mmHg, and one study suggests adipose tissue-derived MSCs are inherently hypoxia tolerant, with oxygen levels as low as 1% having no effect on survival [21]. Indeed, in the present study, the viability of hADSCs was not affected even under nitrogen gas replacement conditions, suggesting that hypoxia itself not only inhibits cell aggregation but also poses a low risk to the cells.

On the other hand, studies with vascular endothelial cells and human umbilical vein endothelial cells have shown that exposure to reactive oxygen species (ROS) results in altered expression of cell surface proteins and increased adhesion [22,23]. Although it remains unclear which factors mediate the effects of ROS on cell adhesion, lowering the pO_2_ in the suspension may suppress ROS generation. Perhaps the three preservation conditions presented here suppress the generation of ROS by lowering pO2 and inhibit the aggregation of the preserved cells by controlling changes in the expression of cell surface proteins.

## 5 Conclusions

In the present study, high cell aggregation rates were observed when hADSCs were stored in LR-3T-5D for 24 h at 25 °C, although their viability was maintained. Our data suggest that cell aggregation can be reduced by lowering the pO_2_ of the preservation solution by increasing the volume and cell density as well as by nitrogen gas replacement.

## Declaration of competing interest

The experimental work presented in this article was performed while T. Kikuchi, M. Nishimura, C. Shirakawa, and Y. Fujita were employed at Otsuka Pharmaceutical Factory, Inc. T. Otoi has no financial interest. Non-financial interest: none.
